# What core primary health care services should be available to Australians living in rural and remote communities?

**DOI:** 10.1186/1471-2296-15-143

**Published:** 2014-08-21

**Authors:** Susan L Thomas, John Wakerman, John S Humphreys

**Affiliations:** 1 Centre for Remote Health, Flinders University and Charles Darwin University, PO Box 4066, Northern Territory 0871, Alice Springs, Australia; 2 Centre of Research Excellence in Rural and Remote Primary Health Care, Bendigo, Australia; 3 Flinders Northern Territory, Darwin, Australia; 4 School of Rural Health, Monash University, Bendigo, Australia

**Keywords:** Primary health care, Equity, Access, Core services, Health service planning, Health policy, Rural, Remote

## Abstract

**Background:**

Australians living in rural and remote areas experience poorer access to primary health care (PHC) and poorer health outcomes compared to metropolitan populations. Current health reform in Australia aims to ensure all Australians, regardless of where they live, have access to essential PHC services. However, at a national level policy makers and health planners lack an evidence-based set of core PHC services to assist in implementing this goal.

**Methods:**

A Delphi method was used to reach consensus on an evidence-based list of core PHC services to which all Australians should have access and their necessary support functions. Experts in rural and remote and/or Indigenous PHC, including policy-makers, academics, clinicians and consumers, were invited to consider a list of core services derived from the literature.

**Results:**

Thirty nine experts agreed to participate. After three survey rounds there was a strong consensus (≥80% agreement) on core PHC services namely; ‘care of the sick and injured’, ‘mental health’, ‘maternal/child health’, ‘allied health’, ‘sexual/reproductive health’, ‘rehabilitation’, ‘oral/dental health’ and ‘public health/illness prevention’; and on the PHC support functions of; ‘management/governance/leadership’, ‘coordination’, ‘health infrastructure’, ‘quality systems’, ‘data systems’, ‘professional development’ and ‘community participation’. Themes emerging from qualitative data included challenges in providing equitable PHC in rural and remote areas, the importance of service coordination and diverse strategies to overcome access barriers.

**Conclusion:**

This study identifies a basket of PHC services that consumers in rural and remote communities can expect to access. It provides rigorously derived evidence that will contribute to a more systematic approach to PHC service planning and availability and will assist policy makers in the allocation of scarce resources necessary to improve the health outcomes of residents of rural and remote areas.

## Background

People living in rural and remote areas in Australia experience poorer access to health care services, exhibit a higher prevalence of health risk factors and greater rates of illness, hospitalisation and death compared to metropolitan populations. These health outcomes generally worsen with distance from capital cities [1]. Other developed countries such as Canada and the United States experience similar health disparities between rural and remote populations and those living in metropolitan areas [2]. Poorer access to primary health care (PHC) in rural and remote areas, due to a lack of necessary infrastructure and workforce, contributes to poorer health outcomes [3].

Primary health care (PHC) is an effective and efficient model for providing a range of basic health services and access to essential PHC services is a factor in improving health outcomes [4]–[7]. Primary Health Care refers to “… *socially appropriate, universally accessible, scientifically sound first level care provided by health services and systems with a suitably trained workforce comprised of multi-disciplinary teams supported by integrated referral systems in a way that: gives priority to those most in need and addresses health inequalities; maximises community and individual self-reliance, participation and control; and involves collaboration and partnership with other sectors to promote public health. Comprehensive primary health care includes health promotion, illness prevention, treatment and care of the sick, community development, and advocacy and rehabilitation”*[8]*.*

Many countries, including Australia, are undertaking significant health reform with a major commitment to PHC as a means of providing equitable health care for all that is accessible, effective and sustainable [9,10]. Australia recently released its first national PHC strategy (*Building a 21st Century Primary Health Care System; Australia’s First National Primary Health Care Strategy, 2010*). It aims to ensure PHC services are better able to respond to local needs, and to provide a coordinated, comprehensive service that functions to promote health, prevent illness and reduce the current over-reliance on hospital services [11]. How best to provide equitable access to PHC services in rural and remote areas of Australia is therefore a central policy issue.

It follows then that a key question for policy-makers is “*what are the core PHC services that should be available to all Australians, regardless of where they live*”? Currently policy-makers and health service planners charged with the responsibility for allocating health resources to rural and remote communities do so in the absence of any comprehensive and agreed national listing of what constitute ‘core’ or essential PHC services. A recent systematic review found no definitive list of ‘core PHC services’. Instead there was wide variability depending on the purpose of the study, methods employed and the setting [12]. Arguably, the current absence of any conclusive national list of core PHC services in Australia contributes to the significant service gaps and inconsistencies in health service planning and is a barrier to reform policy implementation.

This study aimed to provide a template for policy makers and service planners seeking to ensure the equitable allocation of scarce resources. This first stage is to ascertain exactly what PHC services should be available to all Australians, regardless of where they live. Such a list will assist with determining resource allocation and will inform rural and remote consumers of what PHC services they might reasonably expect to access.

## Method

A Delphi technique was used to reach an expert consensus on a list of core PHC services and necessary support functions. The Delphi method employs successive iterations of survey results, whereby the researchers summarise and feed back results between rounds, allowing panellists to reconsider their answers in light of the collective response of their peers. This occurs until a level of saturation or consensus is achieved. Ensuring privacy and confidentiality, the panellist’s identity was known only to the researchers. The anonymity of the Delphi process prevents dominance by some individuals and allows for less popular or less forceful opinions to be considered [13]. This method was considered the most appropriate since the subject of core PHC services and support functions is complex, there is little published literature, and opinions on the topic are diverse. In addition, being able to conduct it using interactive technology, allowed the participation of a wide range of experts from across Australia without them having to meet face-to-face [14,15].

The Delphi group included experts in rural, remote and/or Indigenous PHC. Sixty six potential panellists were identified using a comprehensive list developed in a previous study [16] and with the assistance of a national expert advisory group. Invited experts had at least five years’ experience working in the field of rural or remote health. Particular attention was paid to ensuring wide representation from the areas of policy, academe, clinical practice and consumer representation. All states and territories were represented. Members of key rural and remote health organisations were included. Potential panellists received a participant information statement with a letter of invitation. Informed consent was implied as panellists completed the first survey.

The survey instrument was developed using the references from the systematic review previously cited [12]. To develop the list of core PHC services and functions we reviewed all papers cited in the recent systematic review and selected those relevant to the Australian context (that is, for a ‘high income country’ rather than a ‘medium or low income’ country). We re-read those papers and reports, extracting the PHC services and functions put forward as necessary or appropriate. The research team collated and grouped these in broad categories. We listed PHC services rather than professional categories (access to physiotherapy for example, not to physiotherapists) or service models (such as outreach or home visiting). As most relevant to the rural and remote context, we drew largely on Australian literature including but not limited to publications developed for Aboriginal and Torres Strait Islander populations. The survey was piloted.

Results from the first round were circulated in the second round. In response to panellists’ initial comments we included a list of examples (illustrative lists) of each of the core services and functions. We developed these illustrative lists by reviewing the papers form the systematic review and selecting examples of the sorts of services and functions that may be included under the broad categories. These lists were not meant to be exhaustive nor prescriptive and panellists were advised that local services needed to be tailored to meet the needs of the community. For the purpose of this Delphi study, ‘services’ refer to prevention, detection, treatment and rehabilitation provided to patients, families and communities while ‘functions’ support the provision of those health services [17].

Using a Likert scale (‘strongly agree’, ‘agree’, ‘neither agree nor disagree’, ‘disagree’ or ‘strongly disagree’), Delphi panellists scored each of the core PHC services that all Australians should be able to access, each of the support functions necessary to ensure sustainable PHC services, and all the examples on the illustrative lists. Panellists were asked not to prioritise services or to answer according to current workforce, infrastructure or fiscal constraints, but rather to consider what PHC services they believe should be available to ensure good health based on their understanding of rural and remote health needs. A Delphi survey is usually considered complete when there is a consensus of opinion or when some point of diminishing returns is reached. In the absence of any firm rules defining consensus [18], we used the following:

• Strong consensus- ≥80% of panellists either ‘agree’ or ‘strongly agree’

• Moderate consensus- 60-79% of panellists either ‘agree’ or ‘strongly agree’

• No consensus- < 60% of panellists either ‘agree’ or ‘strongly agree’

Panellists could comment on the individual services and functions and provide any additional general comments. Comments were extracted from each survey round and grouped by the study authors into themes. Consideration was given to the number of participants who put forward similar comments and their relevance to the study aims. Analysis was done manually, initially by the lead author and then with the participation of co-authors. Agreed themes were included in successive survey iterations for consideration by the panellists. The themes reported in this paper are those that developed over all the Delphi survey rounds.

Surveys were developed and implemented using Survey Monkey^®^ and emailed to panellists. Three iterations were completed between August and December 2012. Results were analysed using Microsoft Excel 2010^®^.

Ethics approval was obtained from the Central Australian Human Research Ethics Committee (CAHREC 12–57).

## Results

A total of 66 experts were invited to participate and 39 accepted. Table 1 shows the categories of Delphi panellists who accepted the initial invitation and the response rates for the three rounds. There was an even gender distribution and experts were located in all states and Territories. All 39 panellists were invited to complete round one and two. Only participants who completed round two were invited to complete round 3 as those who completed only round one had not contributed to the consensus process on the illustrative lists introduced in round two. There was a strong or moderate consensus for all services and functions.

**Table 1:** Category of Delphi panellists with response rates for three survey iterations

**Category of panellists expertise**	**Accepted invitation to participate and sent round 1 survey**	**Responded to round 1 n (%)**	**Sent round 2 survey**	**Responded to round 2 n (%)**	**Sent round 3 survey**	**Responded to round 3 n (%)**
Policy/management	16	12 (75.0)	16	11 (68.8)	11	11 (100)
Clinician	8	8 (100)	8	7 (87.5)	7	6 (85.7)
Academic	11	9 (81.8)	11	9 (81.8)	9	7 (77.8)
Consumer representative	4	4 (100)	4	4 (100)	4	4 (100)
**Total**	**39**	**33 (84.6)**	**39**	**31 (79.5)**	**31**	**28 (90.3)**

After two rounds there was a strong consensus on all PHC core services and functions (Table 2).

**Table 2:** Second iteration; Delphi consensus on core primary health care services and necessary support functions

**Core Primary Health Care Services**	**Strongly agree**	**Agree**	**Neither agree nor disagree**	**Disagree**	**Strongly disagree**	**Total**	**Agree or strongly agree**^**1**^
	**n (%)**	**n (%)**	**n (%)**	**n (%)**	**n (%)**	**n (%)**	**n (%)**
**Care of the Sick and Injured**	29 (93.5)	1 (3.2)	1 (3.2)	0 (0)	0 (0)	31 (100)	30 (96.8)
**Mental Health/Social and Emotional Well Being**	25 (80.6)	4 (12.9)	2 (6.5)	0 (0)	0 (0)	31 (100)	29 (93.5)
**Maternal and Child Health**	24 (77.4)	6 (19.4)	1 (3.2)	0 (0)	0 (0)	31 (100)	30 (96.8)
**Allied Health**	18 (58.1)	10 (32.3)	2 (6.5)	1 (3.2)	0 (0)	31 (100)	28 (90.3)
**Sexual and Reproductive Health**	17 (54.8)	10 (32.3)	3 (9.7)	1 (3.3)	0 (0)	31 (100)	27 (87.1)
**Rehabilitation**	13 (41.9)	13 (41.9)	3 (9.7)	2 (6.5)	0 (0)	31 (100)	26 (83.9)
Oral/Dental Health*	23 (76.7)	6 (20.0)	1 (3.3)	0 (0)	0 (0)	30 (100)	29 (96.7)
**Public Health/Illness Prevention**	24 (77.4)	6 (19.4)	0 (0)	1 (3.2)	0 (0)	31 (100)	30 (96.8)
**Necessary Support Functions**							
**Management/Governance/Leadership**	22 (71.0)	9 (29.0)	0 (0)	0 (0)	0 (0)	31 (100)	31 (100)
**Coordination**	27 (87.1)	4 (12.9)	0 (0)	0 (0)	0 (0)	31 (100)	31 (100)
**Health Infrastructure**	18 (58.0)	10 (32.3)	2 (6.5)	1 (3.2)	0 (0)	31 (100)	28 (90.3)
**Quality Systems**	21 (67.7)	9 (29.0)	1 (3.2)	0 (0)	0 (0)	31 (100)	30 (96.8)
**Data Systems**	20 (64.5)	9 (29.0)	2 (6.5)	0 (0)	0 (0)	31 (100)	29 (93.5)
**Professional Development**	20 (64.5)	9 (29.0)	2 (6.5)	0 (0)	0 (0)	31 (100)	29 (96.8)
**Community Participation**	23 (74.2)	6 (19.4)	2 (6.5)	0 (0)	0 (0)	31 (100)	29 (93.5)

^1^≥80% agree or strongly agree indicates strong consensus.

*one missing value.

A strong consensus was achieved for all PHC services and functions. For core PHC services the highest level of consensus (96.8%) was achieved for ‘care of the sick and injured’, ‘maternal and child health’ and ‘public health/illness prevention’. ‘Oral/dental health’ also recorded a high level of consensus (96.7%). While still a strong consensus, ‘rehabilitation’ recorded 83.9% either agreeing or strongly agreeing. For the support functions the level of consensus was consistently above 90% with ‘management/governance/leadership’ and ‘coordination’ attaining a consensus of 100%.

In the third round there was consensus on the more detailed illustrative lists of services (Table 3) and functions (Table 4).

**Table 3:** Third iteration; Delphi consensus on illustrative lists of core primary health care services

**Core Primary Health Care Services**	**Strongly agree**	**Agree**	**Neither agree nor disagree**	**Disagree**	**Strongly disagree**	**Total**	**Strong consensus**^**1**^	**Moderate consensus**^**2**^
	**n (%)**	**n (%)**	**n (%)**	**n (%)**	**n (%)**	**n**	**n (%)**	**n (%)**
**Care of the Sick and Injured**								
24 hour care including evacuation and emergency care	25 (89.3)	3 (10.7)	0	0	0	28	28 (100.0)	
Treatment of injury and poisoning	25 (89.3)	3 (10.7)	0	0	0	28	28 (100.0)	
Pathology	11 (39.3)	13 (46.4)	4 (14.3)	0	0	28	24 (85.7)	
Radiology	11 (39.3)	11 (39.3)	6 (21.4)	0	0	28		22 (78.6)
Provision of essential drugs	24 (85.7)	4 (14.3)	0	0	0	28	28 (100.0)	
Patient advocacy	20 (71.4)	3 (10.7)	5 (17.9)	0	0	28	23 (82.1)	
**Mental Health and Social and Emotional Well Being**								
Counselling	20 (71.4)	6 (21.4)	0	2 (7.1)	0	28	26 (92.9)	
Drug and alcohol treatment	15 (53.6)	13 (46.4)	0	0	0	28	28 (100.0)	
**Maternal and Child Health**								
Ante/post natal care	23 (82.1)	5 (17.9)	0	0	0	28	28 (100.0)	
Child development checks	20 (71.4)	7 (25.0)	1 (3.6)	0	0	28	27 (96.4)	
Immunisation	25 (89.3)	3 (10.7)	0	0	0	28	28 (100.0)	
**Allied Health Services**								
Audiology	12 (42.9)	11 (39.3)	3 (10.7)	2 (7.1)	0	28	23 (82.1)	
Dietetics	10 (35.7)	12 (42.9)	4 (14.3)	2 (7.1)	0	28		22 (78.6)
Occupational therapy	8 (28.6)	16 (57.1)	4 (14.3)	0	0	28	24 (85.7)	
Optometry	15 (53.6)	11 (39.3)	2 (7.1)	0	0	28	26 (92.9)	
Physiotherapy	17 (60.7)	9 (32.1)	1 (3.6)	1 (3.6)	0	28	26 (92.9)	
Podiatry	15 (53.6)	6 (21.4)	7 (25.0)	0	0	28		21 (75.0)
Psychology	17 (60.7)	8 (28.6)	2 (7.1)	1 (3.6)	0	28	25 (89.3)	
Counselling/social work/family violence	20 (71.4)	7 (25.0)	1 (3.6)	0	0	28	27 (96.4)	
Speech pathology	11 (39.3)	11 (39.3)	6 (21.4)	0	0	28		22 (78.6)
Aged care and disability services	18 (64.3)	9 (32.1)	1 (3.6)	0	0	28	27 (96.4)	
Palliative care	17 (60.7)	9 (32.1)	2 (7.1)	0	0	28	26 (92.9)	
**Sexual and Reproductive Health**								
Sexually transmitted infections and blood borne viruses	18 (64.3)	9 (32.1)	1 (3.6)	0	0	28	27 (96.4)	
Family planning	22 (78.6)	5 (17.9)	1 (3.6)	0	0	28	27 (96.4)	
**Rehabilitation**								
After trauma	14 (50.0)	11 (39.3)	3 (10.7)	0	0	28	25 (89.3)	
Post-CVA (stroke)	14 (50.0)	11 (39.3)	3 (10.7)	0	0	28	25 (89.3)	
Alcohol and other drug rehabilitation	13 (46.4)	12 (42.9)	3 (10.7)	0	0	28	25 (89.3)	
**Public Health/Illness Prevention**								
Immunisation	26 (92.9)	2 (7.1)	0	0	0	28	28 (100.0)	
Communicable disease control	23 (82.1)	4 (14.3)	1 (3.6)	0	0	28	27 (96.4)	
Targeted population/health promotional programs	19 (67.9)	7 (25.0)	2 (7.1)	0	0	28	26 (92.9)	
Screening programs	21 (75.0)	7 (25.0)	0	0	0	28	28 (100.0)	
Youth programs	17 (60.7)	8 (28.6)	2 (7.1)	1 (3.6)	0	28	25 (89.3)	
Well men’s and women’s services	18 (64.3)	5 (17.9)	4 (14.3)	1 (3.6)	0	28	23 (82.1)	
Advocacy	15 (53.6)	9 (32.1)	4 (14.3)	0	0	28	24 (85.7)	

^1^≥ 80% ‘agree’ or ‘strongly agree’ indicates a strong consensus.

^2^60-79% ‘agree’ or ‘strongly agree’ indicates a moderate consensus.

**Table 4:** Third iteration; Delphi consensus on illustrative lists of core primary health care support functions

**Core primary health care support functions**	**Strongly agree**	**Agree**	**Neither agree nor disagree**	**Disagree**	**Strongly disagree**	**Total**	**Strong consensus**^**1**^	**Moderate consensus**^**2**^
	**n (%)**	**n (%)**	**n (%)**	**n (%)**	**n (%)**	**n**	**n (%)**	**%**
**Management/Governance/Leadership**								
Human resources management	17 (60.7)	10 (35.7)	1 (3.6)	0	0	28	27 (96.4)	
Human resources management	15 (53.6)	12 (42.9)	1 (3.6)	0	0	28	27 (96.4)	
Transparent systems of accountability	22 (78.6)	6 (21.4)	0	0	0	28	28 (100.0)	
Advocacy at an organisational, regional and potentially national level	19 (67.9)	5 (17.9)	4 (14.3)	0	0	28	24 (85.7)	
Formulating service policy and service planning at all levels	17 (60.7)	9 (32.1)	1 (3.6)	1 (3.6)	0	28	26 (92.9)	
**Coordination**								
Hospital liaison/discharge planning	26 (92.9)	2 (7.1)	0	0	0	28	28 (100.0)	
Linkages with other health and community services	24 (85.7)	4 (14.3)	0	0	0	28	28 (100.0)	
Coordination across related sectors	13 (46.4)	12 (42.9)	3 (10.7)	0	0	28	25 (89.3)	
Partnership between clinical and public health services with a focus on PHC	21 (75.0)	7 (25.0)	0	0	0	28	28 (100.0)	
**Health Infrastructure**								
Buildings, materials, systems of maintenance	21 (75.0)	4 (14.3)	3 (10.7)	0	0	28	25 (89.3)	
**Quality Systems**								
Evidence based practice	20 (71.4)	7 (25.0)	1 (3.6)	0	0	28	27 (96.4)	
Monitoring and evaluation	21 (75.0)	7 (25.0)	0	0	0	28	28 (100.0)	
Quality improvement systems	22 (78.6)	4 (14.3)	2 (7.1)	0	0	28	26 (92.9)	
**Data Systems**								
Health records, data collection, public health data collection, monitoring and follow up systems, health registers	25 (89.3)	2 (7.1)	1 (3.6)	0	0	28	27 (96.4)	
**Professional Development**								
Training, support, supervision, preparing staff for rural and remote contexts and multi-disciplinary team practice	23 (82.1)	4 (14.3)	0	1 (3.6)	0	28	27 (96.4)	
**Community Participation**								
Promoting cultural safety	20 (71.4)	7 (25.0)	1 (3.6)	0	0	28	27 (96.4)	
Ensuring service responsiveness	21 (75.0)	7 (25.0)	0	0	0	28	28 (100.0)	
Contributing to good governance	20 (71.4)	6 (21.4)	2 (7.1)	0	0	28	26 (92.9)	

^1^≥ 80% ‘agree’ or ‘strongly agree’ indicates a strong consensus.

^2^60-79% ‘agree’ or ‘strongly agree’ indicates a moderate consensus.

A strong consensus was achieved for most of the services on the illustrative lists of services. There was a 100% consensus for ‘24 hour care including evacuation and emergency care’, ‘treatment of injury and poisoning’, ‘provision of essential drugs’, ‘drug and alcohol treatment’, ‘ante/post natal care’, ‘immunisation’ (as part of both ‘maternal and child health’ and ‘public health /illness prevention’) and also for ‘screening programs’. While still a strong consensus, rates were lower (82.1%) for ‘patient advocacy’ (part of ‘care of the sick and injured’), ‘audiology’ and ‘well men’s and women’s services’. A moderate consensus was reached for a small number of services on the illustrative lists. They included ‘radiology’, ‘dietetics’, ‘speech pathology’ (78.6%) and ‘podiatry’ (75.0%). The broad category of ‘oral/dental’ health did not include illustrative lists of what may be included here and therefore does not appear in Table 3.

There was strong agreement for all of the support functions on the illustrative lists with 100% consensus for ‘transparent systems of accountability’, ‘hospital liaison/discharge planning’, ‘linkages with other health and community services’, ‘partnerships between clinical and public health services’, ‘monitoring and evaluation’ and ‘ensuring service responsiveness’.

### Themes from panellist’s comments

In addition to completing the Likert Scales, most panellists contributed at least one comment during the surveys. In round one 29/39 (88%) contributed at least one comment, in round two 23/39 (80%) and in round three 14/28 (50%) did so.

Three main themes emerged from panellists’ comments: *i*) the inherent challenges of providing equitable PHC in rural and remote areas; *ii*) the importance of service coordination; and *iii*) the diverse ways to overcome access barriers.

Some of the inherent challenges in service provision included difficulties in recruiting and retaining a skilled workforce, maintaining and extending health infrastructure, ensuring service quality and safety with a small population base, and ensuring affordability. Other challenges included overcoming barriers imposed by distance and time, particularly when health needs were urgent. There was a sense that even though it may be difficult to provide a full range of PHC services locally, it was important to ensure ready access to them.

‘Priorities are always an unfortunate part of providing health services in rural and remote Australia….it is the exclusion or downplaying of services that creates problems and hurts communities.’ (Consumer Representative)

Specialised services such as radiology/pathology will need a population base to provide a quality/safe service (Clinician)

Secondly, panellists commented on the importance of service coordination. Examples included follow-up care and providing more complex care for those with chronic illness. Coordination of services between small communities and larger centres was important to ensure individuals don’t ‘slip through the gap’. Other examples related to inter-sectoral collaboration with departments of housing, education and environmental services, which addressed the social determinants of health.

‘[There is] a major issue about chronic otitis media in Aboriginal communities where even though a diagnosis is made, there is little follow-up, little attention to addressing the social determinants and as a result continued loss of hearing in children with consequent learning difficulties and other sequelae.’ (Academic)

‘Addressing the upstream determinants of heath seems essential if health and wellbeing [is] the ultimate goal.’ (Consumer Representative)

Finally, panellists noted the many different ways in which PHC services can be provided to overcome access barriers, including the use of tele-health and tele-radiology strategies. Others focused on local health workers with multiple generalist skills, supported by regular visiting specialists. This included ‘fly-in, fly-out’, ‘hub-and-spoke’, periodic outreach and other similar regional approaches. It was important to support patients with access to 24 hour transport assistance and to ensure that the frequency of visiting services and support was commensurate with community needs.

‘My answers do not imply that such services should be located in every community but there should be reasonable and convenient access (including electronic access where appropriate) depending on the seriousness and urgency of the situation.’ (Policy/Management)

‘…expansion of some allied health services through tele-health/up skilling of PHC professionals based in communities should increase access.’ (Policy/Management)

## Discussion

In considering what core PHC services should be available to all Australians regardless of where they live, there was a particularly strong consensus for ‘care of the sick and injured’, ‘maternal and child health’, ‘oral/dental health’ and ‘public health/illness prevention’ services. This may reflect the *urgent care* needs of many underserviced rural and remote communities, the persistent gaps in accessibility, and possibly the vulnerability of those in need. Support of ‘public health/illness prevention’ reflects recognition of the importance of services that have a *whole-of-population* effect, that focus on prevention and early detection of health problems and can address the social determinants of health. The moderate consensus for ‘radiology’ and for some of the ‘allied health services’ (‘dietetics’, ‘podiatry’ and ‘speech pathology’) may reflect assumptions that these services required more technical skills or that identified current workforce constraints discriminate against equitable access. While a small number indicated that they ‘neither agreed nor disagree’ and an even smaller number chose to ‘disagree’, consensus was still ≥ 75%.

In relation to the support functions, a strong consensus was reached on all the broad categories and all the illustrative lists, particularly ‘management/governance/leadership’, ‘co-ordination’, and the illustrative examples that described ‘transparent systems of accountability’, liaison, linkages and partnerships with other health agencies, ‘monitoring, evaluation’ and ‘ensuring service responsiveness’. Panellists commented on the important role these functions play in the integration and co-ordination of services, and that the core services without underlying support functions are insufficient in responding to the health needs of communities. This is consistent with previous research demonstrating the essential requirements for sustaining PHC services in rural and remote areas [19].

For consumers, this study has identified a set of core PHC services which experts considered they should be able to access regardless of where they live. Many residents of rural and remote communities do not currently have ready access to these PHC services, and have come to accept this poor access, characterising the status quo, as normal. Indeed, many rural and remote residents may not even be aware of the range and type of basic PHC services that are necessary for and available to those living in metropolitan areas.

For policy makers and health planners, this list of core PHC services can be used as a guide to identify service gaps and inconsistencies, and to plan appropriate, consistent and effective health workforce and infrastructure strategies necessary to address the health needs of rural and remote Australians. Without a defined and agreed set of core PHC services, resource allocation is likely to be ‘*ad hoc*’ or based on ‘historical’ expenditure, with access to core PHC services remaining ‘patchy’ and inconsistent across similar communities. For health service planners, this ‘basket of services’ can be used to systematically identify health service gaps, take into account community diversity, felt needs and reliable prevalence data, in order to a tailor a specific ‘package’ of core PHC services that meets the needs of the community [20]. The results of this study may be generalisable to other developed countries such as Canada and the United States, where similar health disparities exist between rural and remote populations and those living in urban areas.

This study also provides essential information for developing health service models to provide comprehensive PHC. While outside the scope of this study, panellists offered suggestions of how to improve access to these services, including use of new technologies and by supporting generalist health workers with visiting specialists.

Arguably most important of all, an evidence-based list of core PHC services and support functions provides a vital template to guide the equitable allocation and distribution of scarce resources towards PHC service provision. For policymakers, this list makes possible the development of resource allocation benchmarks for underserviced communities. This investment in PHC services, particularly illness prevention and health promotion, will reduce national health expenditure by reducing avoidable hospitalisation costs and increasing system sustainability [6,11,21]. The authors are currently investigating the role of population thresholds characterising rural and remote communities in order to ascertain which particular core PHC services can be feasibly located in situ and which may need to be provided either on a visiting basis or via the patient travelling to that service in a larger community.

This study is not without limitations. Delphi group limitations may include selection bias, a small number of iterations and a decline in response rates [15]. There are no set rules determining how many panellists should be included, although 8–10 has been suggested as sufficient to ensure validity [22], with time and cost factors considered [18]. The methodological process adopted in this study was rigorous. We made a particular effort to ensure panellists selected were representative of the diverse rural and remote and/or Indigenous PHC field. Many were well-placed to implement findings or advocate for consumers. Other strengths of the study included: the large size of our Delphi group, the high response rates for the three iterations and the insightful comments made by panellists, indicating their commitment and ongoing engagement with the topic.

## Conclusion

In the absence of any agreed set of core PHC services that should be available to all Australians regardless of where they live, and the support functions necessary to ensure their sustainability, there will continue to be significant gaps for rural and remote residents in accessing health care. Policy responses are unlikely to accord with a systematic approach to ensuring equity of access to health services. Knowing what services should be available helps communities and policy-makers to work together to ensure that they can be delivered in a way that is ‘fit-for-purpose’, rather than some expedient ‘one-coat-fits-all’ approach. Moreover, the evidence from this core PHC study will facilitate a more equitable distribution of scarce health resources and thereby assist PHC policy-makers and service planners to achieve more equitable health outcomes.

## Competing interests

The authors have no competing interests in the conduct of this study or in the preparation of the manuscript.

## Authors’ contributions

JW and JSH conceptualised the research aims and methodology, guided the research process and contributed to the manuscript. JW provided overall supervision of the project. All authors were involved in the design of the questionaries and in the interpretation of the results. SLT developed and administered the surveys and undertook initial interpretation of results. SLT prepared the first draft of the manuscript. All authors read and approved the final manuscript.

## Pre-publication history

The pre-publication history for this paper can be accessed here:

http://www.biomedcentral.com/1471-2296/15/143/prepub
